# Which ship-integrated power system enterprises are more competitive from the perspective of patent?

**DOI:** 10.1371/journal.pone.0252020

**Published:** 2021-05-24

**Authors:** Danyang Li, Xinlai Li

**Affiliations:** 1 Research Center for Chinese Science Evaluation, Wuhan University, Wuhan, China; 2 School of Information Management, Wuhan University, Wuhan, China; Universita degli Studi del Molise, ITALY

## Abstract

By analyzing the relevant patent data, the technological competitiveness of enterprises can be objectively evaluated, and the research and development priorities and the technological advantages and disadvantages of each enterprise in the same field can be compared and analyzed. It is considered to be helpful in judging the patent strategy, innovation ability, and the innovation level of enterprises, which is of great practical significance. Based on the field of the ship-integrated power system as an example, considering the Derwent patent holder code, draw the integrated technical strength of ships in the field of the distribution power system; using the ideas of the Boston matrix to measure the field has the competitive advantage of enterprises; by using the social network analysis method for each enterprise, the core technology of mining, based on the S curve of the technology life cycle, analyzes the enterprise concentration each time in order to predict the future trend of development. It can be found that China Shipbuilding Industry Corporation has the largest number of patents, followed by Fuji Electric Co. Ltd, International General Electric, Daewoo shipbuilding ocean engineering Co. Ltd and so on. Considering the patent quantity and the patent quality, the dominant and productive enterprises in this field can be distinguished. The ship-integrated power system and its related core technologies have been rapidly growing at present. The related technology patents will increase rapidly in the next few years, and more and more enterprises will participate in this field.

## 1. Introduction

The ship-integrated power system refers to the combination of the traditional independent mechanical propulsion system and the electric power system in the form of electric energy, which provides electric energy for ship propulsion, communication and navigation, and special operations and daily equipment by means of the electric power network, realizing the comprehensive utilization of the whole energy of ships. The idea of an integrated power system is based on reducing the total cost of future ships and optimizing the composition of ships and systems and equipment. Its design idea is to highlight systematization, integration and modularization. The concrete realization of the ship platform is to provide the energy required by the whole ship in the form of electric power and unify the dispatch, distribution, and management [1, 2].

The ship-integrated power system is the development direction of the ship power and is called another revolutionary leap in the history of shipbuilding technology. The developed countries represented by the United States and Britain took the lead in introducing the concept of ship-integrated power system, and actively carried out research, test and application to ships. Meanwhile, Italy, France and Germany have been promoting the development of research and application in ship-integrated power system [3]. At the beginning of this century, ship-integrated power system first began to be applied in civil ships, mainly in the form of medium-voltage and low-voltage alternating current, such as Atlantis scientific research ship, Oasis of the Seas cruise ship and Dutch Blue Marlin semi-submersible ship [3]. According to the development of ship-integrated power system, the integrated power system can be divided into the first generation and the second generation. The technology of the first generation integrated power system is mature, but it has some disadvantages, such as large equipment volume and weight, low system efficiency and low power supply continuity. The second generation of integrated power system adopts medium voltage direct current system, which breaks through the system frequency limit, reduces the requirement of prime mover speed regulation characteristic, greatly reduces the equipment volume and weight, improves the system efficiency and power supply continuity, but the technical development is not yet mature [4].

Electric propulsion ship, as a new energy ship, is in line with the common global demands for environmental protection at the present stage. The integrated all-electric propulsion system is a major reform in the field of ship power plant. At present, all countries are actively carrying out the research on the integrated all-electric propulsion system and have formulated corresponding development plans. According to the latest “Naval Power and Energy System Technology Development Roadmap”, the United States will advance the development of a new generation of integrated power systems in two phases. At present, ac electric propulsion, superconducting electric propulsion, fuel cell propulsion and integrated all-electric propulsion are the key technological development directions in this field [5, 6]. With the development of electric propulsion ship technology, integrated power system will be the development direction of ship industry in the future. In the face of the increasingly fierce competition in the ship market, companies in the shipbuilding industry must have a clear understanding of the technological status quo and competitive pattern in this field if they want to establish a firm foothold in the global market. At present, the related researches mainly focus on the technical characteristics and application evaluation of the ship-integrated power system, and lack the discussion on the subject from the perspective of the institution around the patented technology.

As an important carrier of independent innovation achievements of innovation subjects, the patented technology is considered more enlightening and close to the commercial application than academic papers, technical reports, and other scientific and technological achievements, and it can more accurately reflect the ability and the level of invention and creation of knowledge- and technology-producing subjects [7]. Therefore, the analysis of the relevant patent data can objectively evaluate the technological competitiveness of enterprises, compare and analyze the R&D objectives and the technological advantages and disadvantages of each enterprise in the same field, and help to judge the patent strategy, innovation ability, and the level of enterprises, which is of great practical significance.

At present, there are many studies on enterprise competitiveness based on patent analysis, which primarily focus on the following three aspects. The competitiveness of an enterprise is analyzed based on the patent measurement, such as Chery Automobile [8], Huawei Company [9], Lenovo Group [10], etc. The evaluation method of the patent-based enterprise technological competitiveness includes a single index [11], multiple index [12], patent portfolio method [13], weighted index [14], etc. Based on the patent measurement, the patent strategy of an enterprise is analyzed, and suggestions for improvement are reported. Based on the analysis of patent intelligence, this paper makes a statistical analysis of the number of patents filed by patentees and excavates the main technical forces. Then, it analyzes the technological competitiveness of enterprises by using the patent technology combination method. Finally, it measures the concentration of enterprises based on the distribution of the life development cycle in order to judge the technological development level and the development trend of each enterprise.

Considering the field of the ship-integrated power system [15] as an example, this paper analyzes the patent technology of the ship-integrated power system for understanding the process of technology development and analyzing the technological competitiveness and the development trend of enterprises in order to provide a reference to researchers in the next step of technical analysis methods.

## 2. Data and methods

The data source of this paper is the Derwent innovation index (DII), which uses English words related to the ship-integrated power system as keywords to carry out an accurate retrieval, adopts the exhaustive retrieval strategy and makes adjustments according to the characteristics of the database, and improves the comprehensiveness of the retrieval results by using truncation symbols. Among them, the following three categories of keywords are used in the English search. Category A uses two keywords related to ships, A1 “ship” and A2 “Marine.” Category B uses 12 keywords related to the integrated power system. Category C uses 9 keywords related to system management. A total of 1349 valid records were retrieved, and the above data were used for an in-depth analysis.

This paper applies the patent econometric analysis method and presents the research results in a visual mode. As an important method of the patent analysis, the patent measurement can describe and display the laws and characteristics of technological innovation in a quantitative way and can help decision-makers better grasp and predict the development laws and trends in the field of technological innovation [16].

The research methods used in this paper include the social network analysis, the Boston matrix model, and the life cycle analysis. The social network analysis is based on the co-occurrence matrix of objects, which can depict the co-occurrence relation network of objects in order to explore the maturity, structure, and the scale of network relations. In this study, the GN algorithm is selected to cluster the IPC classification number of each institution in order to find the core technology of an enterprise. The Boston matrix is a method of planning the product portfolio of a firm, which is pioneered by the Boston Consulting Group, a big American business consultancy. The idea of the Boston matrix is used to measure the patentee with a competitive advantage in the field of the integrated power system. The S curve of the technology life cycle is defined as the development of some parameters or the performance of the technology system in accordance with the S-curve law. In this paper, the technology life cycle is divided based on the S curve, and the enterprise concentration in each period is analyzed in order to predict the future development trend.

## 3. Results and discussion

### 3.1. Technical strength distribution within the domain

To some extent, the number of patents of an enterprise can reflect its technological innovation and technological strength. In general, the more patent applications or patents an enterprise has, the stronger its technical competitiveness is. Therefore, competitors and partners can be distinguished by comparing patent data [17, 18].

The DII database provides the patentee code, which is a four-letter code developed by the DII database for each patentee of each patent document filed. There are two types of codes, namely, standard codes and non-standard codes, which are usually determined based on the name of the patentee and represent the same patentee. Considering Derwent’s patentee code, this paper draws the technical strength distribution of de-duplication data. Except in special cases, this paper, by default, considers a patent code as a patentee. This paper with a patentee code of 755, and in accordance with the standard code, non-standard code, and individuals to distinguish, studies enterprise technological competitiveness, and the non-standard code usually consists of multiple institutions, which are not the same, similar to the main institutions in the case of the statistical code for the unit, as shown in Table 1.

**Table 1:** The number of corporate patents (Top 20).

Patent Institution code (standard)	Patent number	Patent Institution code (non-standard)	Patent number
CSHI-C	64	SHAN-Non-standard	46
FJIE-C	52	BEIJ-Non-standard	29
GLDS-C	38	JIAN-Non-standard	24
GENE-C	33	NISH-Non-standard	20
WTNE-C	33	ANHU-Non-standard	16
DEWO-C	32	GUAN-Non-standard	15
USHM-C	29	YICH-Non-standard	14
MITO-C	19	CONV-Non-standard	13
SIEI-C	18	SHEN-Non-standard	13
TOKE-C	18	UYJI-Non-standard	11
CRRC-C	15	WUXI-Non-standard	10
HHIH-C	15	CHON-Non-standard	9
RORO-C	14	NING-Non-standard	9
UHEG-C	14	ZHEN-Non-standard	9
SMSU-C	13	WUHA-Non-standard	8
UWHT-C	12	CHEN-Non-standard	7
WART-C	10	NANT-Non-standard	7
ALLM-C	9	ZHON-Non-standard	7
HITA-C	9	CNRS-Non-standard	6
YANM-C	9	FOSH-Non-standard	6

According to statistics, there are a total of 415 patent organizations that have patents related to the ship-integrated power system. Table 1 shows that the China Shipbuilding Industry Corporation (CSHI-C) has the largest number of patents (64 patents). It is followed by Japan’s Fuji Electric (FJIE-C), GLDS-C, Britain’s General Electric (GENE-C), WTNE-C, and Daewoo Shipbuilding and Offshore Engineering (DEWO-C). In terms of quantity, the first ten patentee codes represented by the institution may be considered high in the ship-integrated power-system-related technologies.

The non-standard code is not a general name of an institution, but a combination of the patentee with the same first four letters of the patent institution into the same code, so it requires to be analyzed in detail according to the full name of the patentee. However, from Table 1 it can be seen that the code of the non-standard institution with the largest number of patent rights is Shan-non-standard. There are a total of 46 patents related to the ship-integrated power system, including some research institutions and technology companies in Shanghai, China, as well as some institutions in the Shandong province, China.

### 3.2. Technical content distribution within the domain

The database provides the following three patent classification numbers for patent records: International Patent Classification Number (IPC number), Derwent Code Classification Number (DC number), and Derwent Manual Classification Number (MC number). Among them, the IPC classification system adopts the principle that function and application collectively determine the patent classification, with the function as the main element and the application as the auxiliary element.

**Fig 1:**
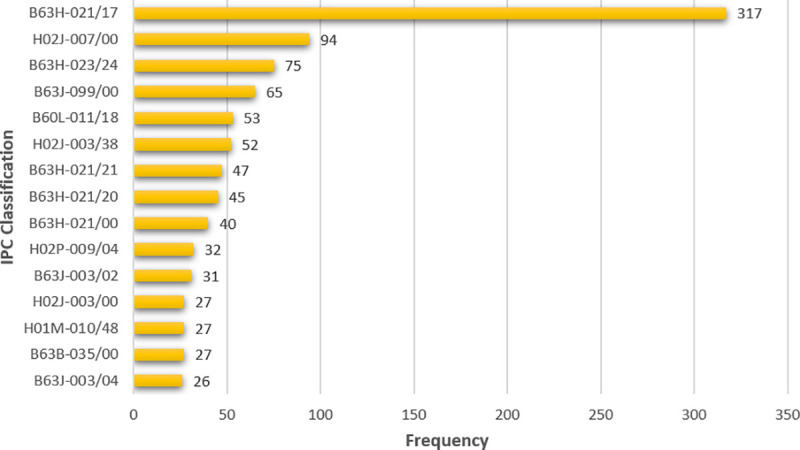
IPC number statistics of deweighting data (part).

**Fig 2:**
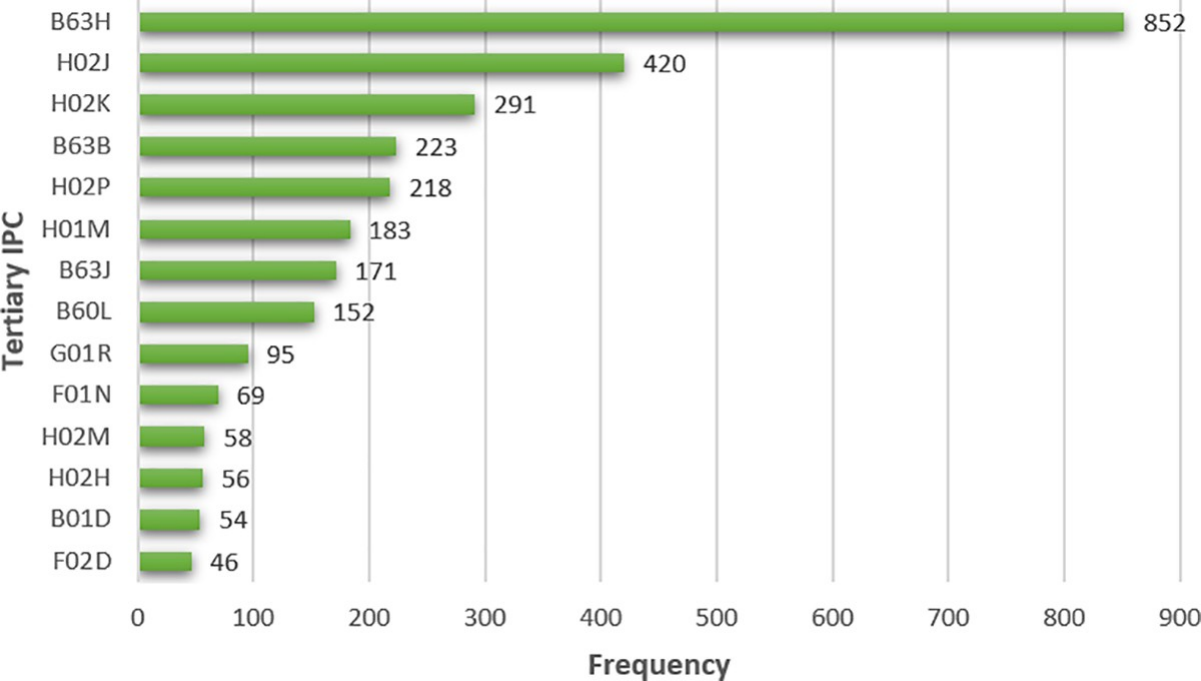
Third-level IPC number statistics of deweighting data (part).

In this paper, a total of 1565 IPC classification numbers appear in 1349 deweighting data, whereas the first 15 high-frequency IPC classification numbers appear in a relatively low frequency and focus on the equipment and devices, digital calculation, control, test, and the detection of the power system (see Fig 1). However, the statistical classification number is a five-level indicator, which describes the patent very carefully. If the statistics are started from the third-level indicator (such as B63H), there are 230 third-level IPC classification numbers, and the first 15 high-frequency third-level classification numbers are shown in Fig 2, primarily focusing on measurement, data processing, electrical device or system, control, and other aspects.

DC classification number is application-oriented and suitable for application-oriented research projects. MC classification number is a supplement to DC classification number. Although exhaustive retrieval strategy is adopted to ensure the full detection rate to some extent, it does not improve the detection rate. In this regard, we select all DC classification numbers to develop the co-occurrence matrix, carry out the condensed subgroup analysis, and obtain the classification number clustering related to the topic.

**Fig 3:**
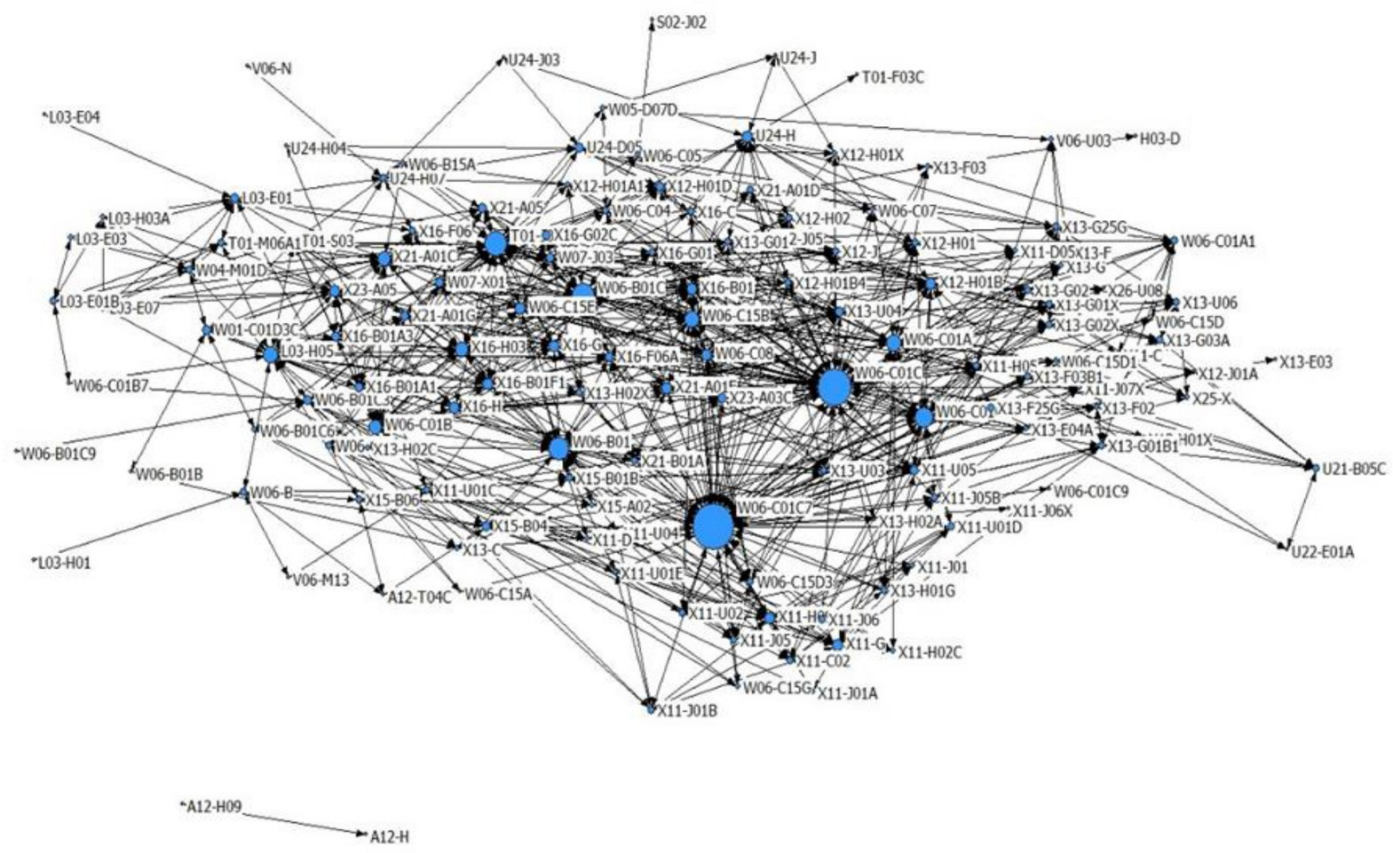
Cluster analysis of the MC number (strong correlation data).

Condensed subgroup is a set of actors that meets the following conditions, that is, the actors in this set have relatively strong, direct, close, frequent or positive relations. Condensed subgroup is used to reveal and characterize substructural states within a population. Figure out the number of condensed subgroups in the patent DC classification number and the specific DC classification number contained in each condensed subgroup, analyze the relation between condensed subgroups and the connection mode, which can investigate the overall situation of patent data from the new dimension. We used CONCOR method with the UCINET software to carry out condensed subgroup analysis. 168 DC classification numbers were divided into 8 clusters based on the co-occurrence matrix of DC classification numbers. Among them, there were 48 DC classification numbers whose frequency was greater than 50. According to the above classification number, the deduplication data were screened, and 453 records with a strong correlation classification number were obtained through cross-comparison with the statistical results of the MC classification number, which were used for the following patent technical analysis (see Fig 3).

### 3.3. Patent technical combination analysis

The concept of the patent portfolio was first proposed by Ernst in 1998, which refers to the patent collection formed around research and development at two different levels in enterprise and technology fields. Patents are often considered as an important indicator of the technical strength of an enterprise. The patent technology portfolio is a dynamic decision-making process leading and organizing the development of patent technology and its results.

Based on the study of patent portfolio behaviors among patentees, the patentees with higher competitive advantages are selected. Considering the existing patent classification system, i.e., International Patent Classification Number (IPC), the social network analysis method is used to realize the subject mining and subject correlation analysis in the field of the integrated power system technology. In this method, NERDRW software is used for visualization. The node represents the IPC classification number, and the size of the node represents the importance of the patent number in the field of the integrated power system technology [16]. If two classification numbers appear in the same patent, it indicates that there is a relationship between the two classification numbers, and the thickness of the line segment can represent the closeness of the relationship between the classification numbers. In the social network analysis, the degree centrality characterizes the core degree of a node. The greater the degree of a node in the network, the more important it is. The core technology of an enterprise can be determined by calculating the centrality of each IPC in the core research area of the enterprise.

In the clustering method of social network, GN algorithm has higher accuracy and better clustering effect on the network with unknown number of categories. The basic idea is to keep deleting the edges in the network that have the largest number of edge interfaces relative to all the source nodes, and repeat the process until all the edges in the network have been removed. In the case of unknown number of clusters, the module function Q is used to measure the partition standard of network modules. Therefore, GN algorithm is selected in this study to cluster the IPC classification number of each institution to find the research field of the institution.

- Enterprise Technology Advantage Measurement

**Fig 4:**
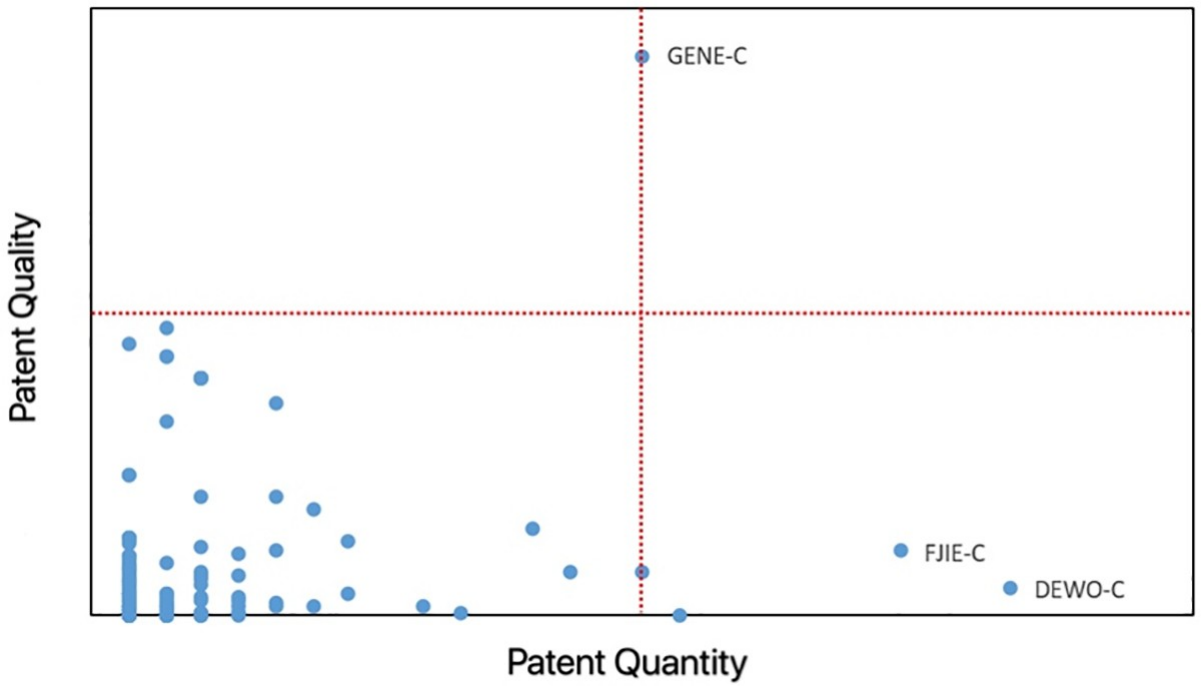
Scatter plot of the total number of patents and the total number of citations at the institution level.

In this study, the idea of the Boston matrix is used to measure the patentees (enterprises) with competitive advantages in the field of the integrated power systems, as shown in Fig 4. It is believed that the patent quantity and the patent quality are two factors that are used to measure the competitive advantage [13]. The patent quantity refers to the number of patents held by an enterprise as the patentee, and the patent quality is the total cited number of patents held by an enterprise as the patentee.

The following four types of enterprises can be formed by combining the above two factors: dominant institutions with both the high patent quantity and the high patent quality, only GENE-C; high-yielding institutions with high patent quantity, but low patent quality, DEWO-C and FJIE-C; high-quality institutions with higher patent quality, but lower number of patents, there is no eligible patentee; inferior institutions with low patent quantity and low patent quality, most institutions belong to this category.

This study focuses on the enterprises of dominant institutions and high-yielding institutions, respectively, and finds out the core technical fields of each institution by subject analysis.

- Core Patented Technology for Dominant Institutions: GENE-C

GENE-C is the code of General Electric Company PLC in the United Kingdom. With a total of 15 patents, GENE-C is a large comprehensive enterprise founded in 1886, with its business scope covering electrical engineering, electronic engineering, communication equipment, defense industry, and other fields. Its headquarter is located in Coventry, United Kingdom.

**Fig 5:**
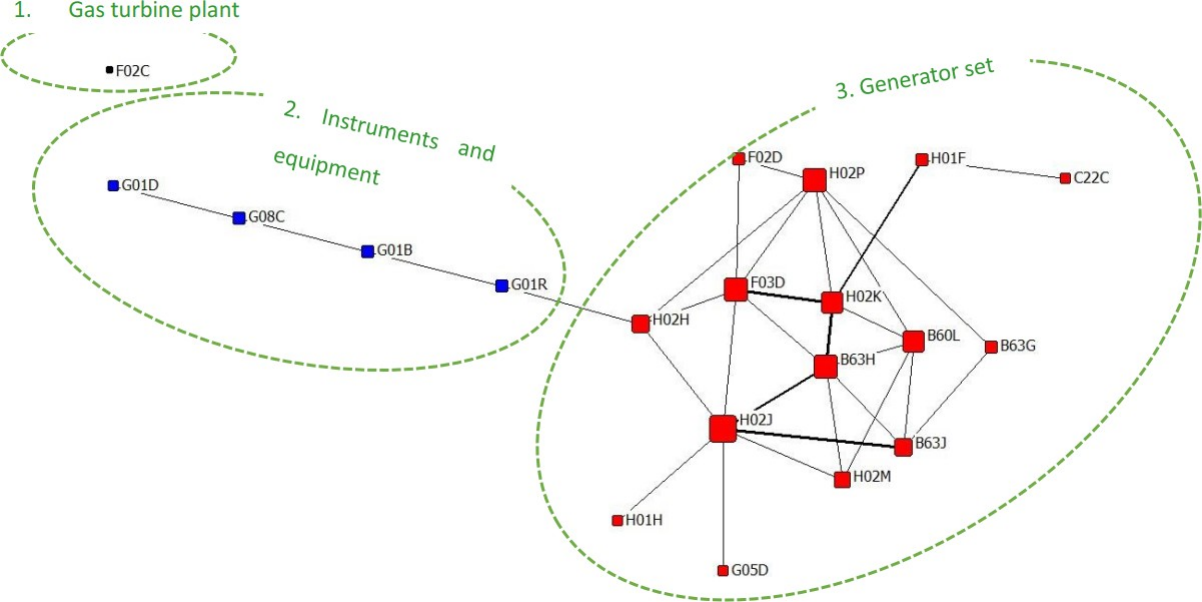
GENE-C patented co-occurrence network diagram.

GE’s patents cover a total of 20 three-level IPC classification number technology areas. A patent can have multiple IPC classification numbers at the same time. The IPC co-occurrence diagram is drawn according to the social network theory, as shown in Fig 5. The node size represents the centrality of IPC, the line segment thickness represents the closeness of IPC connections, and the node color represents the category of IPC (the same as discussed below). According to the category of the GIRVEN-NERMAN cluster, the content in the co-occurrence diagram can be divided into the following three categories.

① Gas turbine modification, which increases the intermediate cooling system; there is a patent.
② The instrument measurement equipment modification, which obtained the sensor in the secondary winding voltage output sum, there is a patent.
③ There are 14 patents for the reconstruction of the generator system. This category is the core of the GE technology, including H02J (36.842), H02P (31.597), F03D (31.597) and B63H (31.597) IPC classifications, and four high centers, involving alternating current into a marine servo system, generator and converter structure design, design modification of the generator stator, and the linear differential pressure sensor and the differential pressure of the rotation sensor.
  - Core Patented Technology for High-yielding Institutions

(1) DEWO-C

DEWO-C is the code name for Daewoo Shipbuilding and Marine Engineering, with 22 patents, which is the world’s second-largest shipbuilding company and one of South Korea’s “big three” shipbuilders.

**Fig 6:**
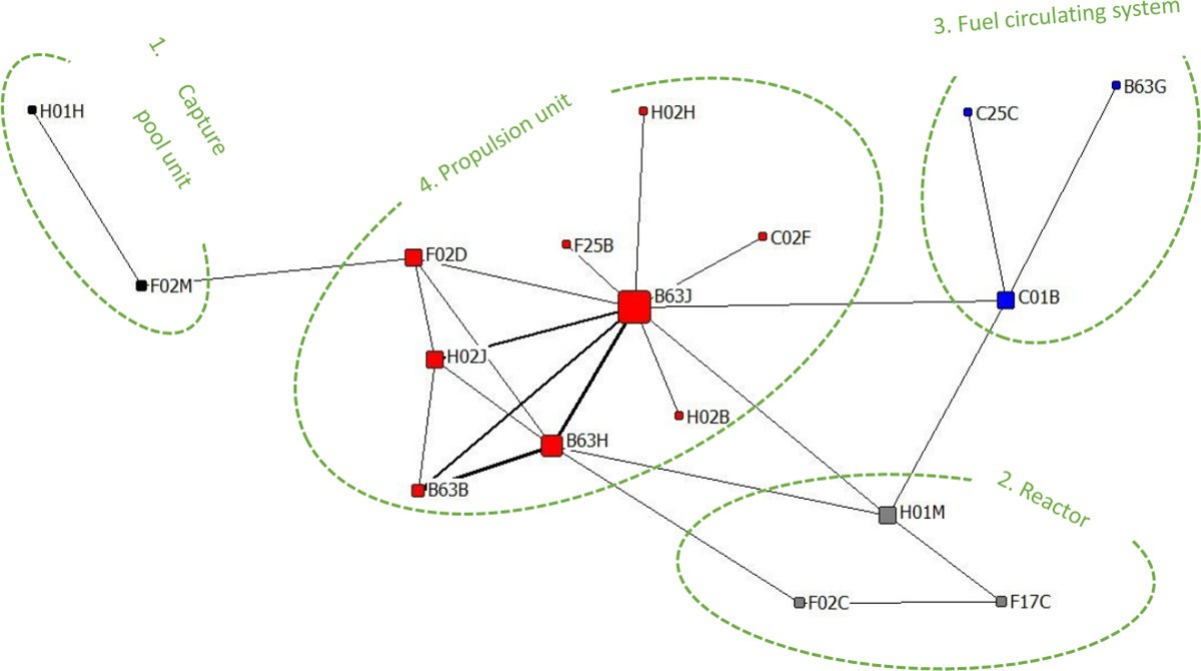
DEWO-C patented co-occurrence network diagram.

Daewoo shipbuilding patents cover a total of 17 three-level IPC classification number fields. A patent can have multiple IPC classification numbers at the same time. The IPC co-occurrence diagram is drawn according to the social network theory, as shown in Fig 6, and the content in the co-occurrence diagram can be divided into the following four categories.

① Capture the pool device, put the fuel oil machine in the capture pool, and add a switch to detect the water level; there is a patent.
② The reactor, which helps to transform the evaporated gas generated in the liquefied natural gas storage tank and to use the steam provided by the steam supply line to produce synthetic gas, which in the solid oxide fuel cell is used to generate electricity by an electrochemical reaction; there is a patent.
③ Fuel circulating system, which improve fuel utilization through the manifold cycles of metallic fuel, while absorption and conversion of harmful components efficiently and reduce pollution; there are three patents.
④ Propulsion device, which increases propulsion system power by adding auxiliary equipment on board; there are 22 patents. It is the core technology field of Daewoo shipbuilding, including B63J (62.500), B63H (37.500), H02J (25.000), and F02D (25.000) with four high degree IPC classification numbers. Among them, it involves the control of the combustion engine, the dual-fuel electric propulsion device, the addition of the horizontal transmitter in the cooling system, the installation of the EGR valve in the circulation pipeline to control the flow of liquefied natural gas (LNG), etc.

(2) FJIE-C

**Fig 7:**
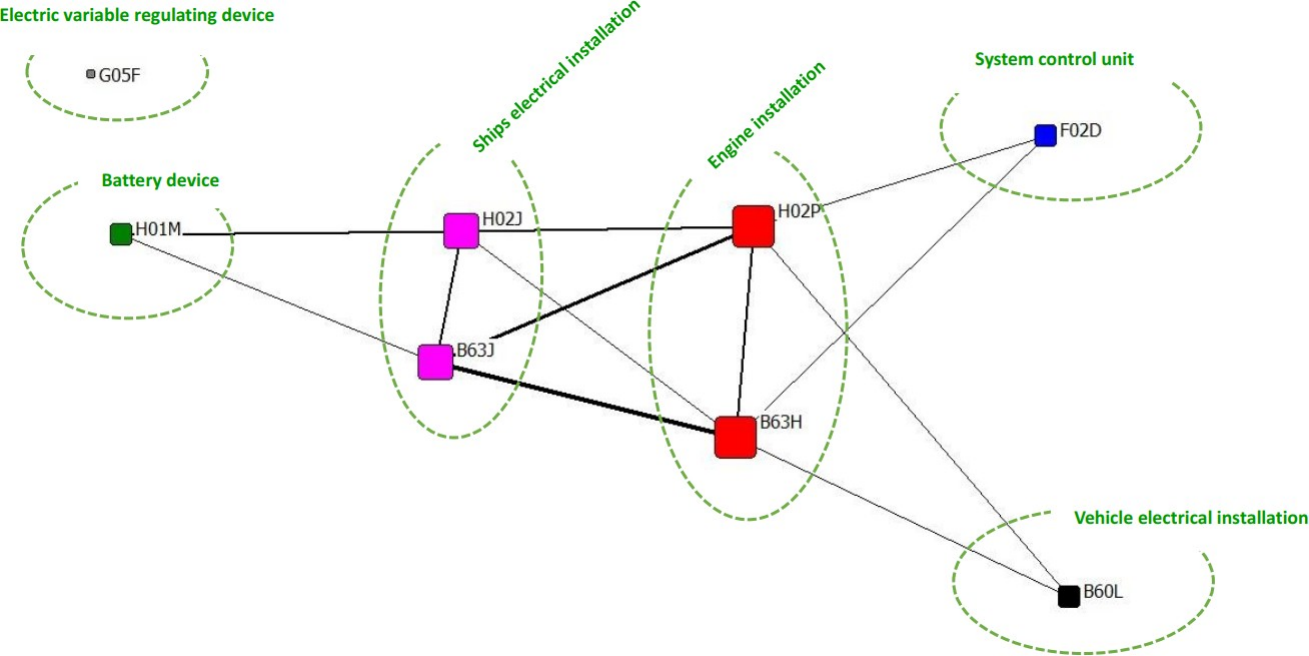
FJIE-C patented co-occurrence network diagram.

FJIE-C is the code for the Fuji Electric Corporation of Japan and holds 22 patents. As shown in Fig 7, the main R&D tasks of the Fuji Electric can be roughly divided into the following six categories.

① Ship propulsion device, which uses the voltage control to improve engine stability; there are 19 patents. The core of the patent is the design of the propulsion system for the ship, which includes the converter that controls the power provided from the power source to the propulsion motor, the rate control unit that controls the power converter, and the operation number of the propulsion motor that can be switched by the control device in order to improve the operation efficiency of the ship. It can effectively accumulate the kinetic energy stored on the ship and improve the safety of the ship during operation.
② Ship circuit device, which improves the efficiency of the charge storage in the ship system; there are 14 patents. The core of the patent is to realize a high-efficiency power supply from the circuit control and the voltage control. The former is an interconnection switch that is designed to open and close the connection between two power supply circuits, thereby suppresses the voltage difference between the batteries, prevents battery, overcharge, characteristic degradation and battery life degradation, and prevents damage to the batteries caused by the impulse current. The latter is the charging power control part that regulates the generator voltage so that the deviation between the actual charging power value and the set charging power value is zero. Thus, it improves the economic effect and the environmental effect of the system and reduces the fuel consumption of the engine and the cost of the generator.
③ System control device, which uses the fuel engine control and improves the operability of the propulsion system; there is a patent. The power converter control circuit and the power converter control the engine to suppress the prime mover turbulence in the ship due to torque.
④ Battery device, which uses the optimized battery charging mode; there are four patents. In addition to the circuit improvement proposed in the second category, a switch between the battery in series and the generator and the chopper used as the power supply is set up in the charging and discharging system in order to bring a decrease in the total charging amount of all batteries and realize the supplementary charging of the characteristic changes of the batteries in series and parallel.
⑤ The electric device of the vehicle and the state monitoring and control device are set; there are two patents. The device is a supplement to the core device, which is called the first-class device. When the power of the generating equipment and the motor exceeds a predetermined fluctuation range, the motor is controlled according to the current power. In the charging and discharging system of the lithium-ion battery of an electric vehicle, a battery charging and discharging state monitoring and control device is set up.
⑥ Electric variable adjustment device, which is a stable drive electric propulsion machinery; it is related to the institution of a patent. The apparatus relates to an initial stage controller circuit. It is mainly used for the execution and traction of the transformer during the initial phase charging circuit and the control of the initial phase charging.

In conclusion, it can be seen that the first two types are the core technologies of the organization. The Fuji Electric Corporation of Japan is committed to improve the ship propulsion system and the control regulation system by using the circuit improvement and voltage control, and thus to improve the effective use of energy and optimize the ship-related devices. According to the thickness of the line segment in Fig 6, it can be seen that the propulsion system of the ship is improved by using electric devices, and the circuit system is optimized by using battery devices, which are closely combined. The technical feature of the agency is that this is a good reason for a company to maintain a steady increase in the number of patents it holds over the course of the development of ship propulsion systems.

### 3.4. Enterprise technology concentration based on life cycle

- Life Cycle Distribution

**Fig 8:**
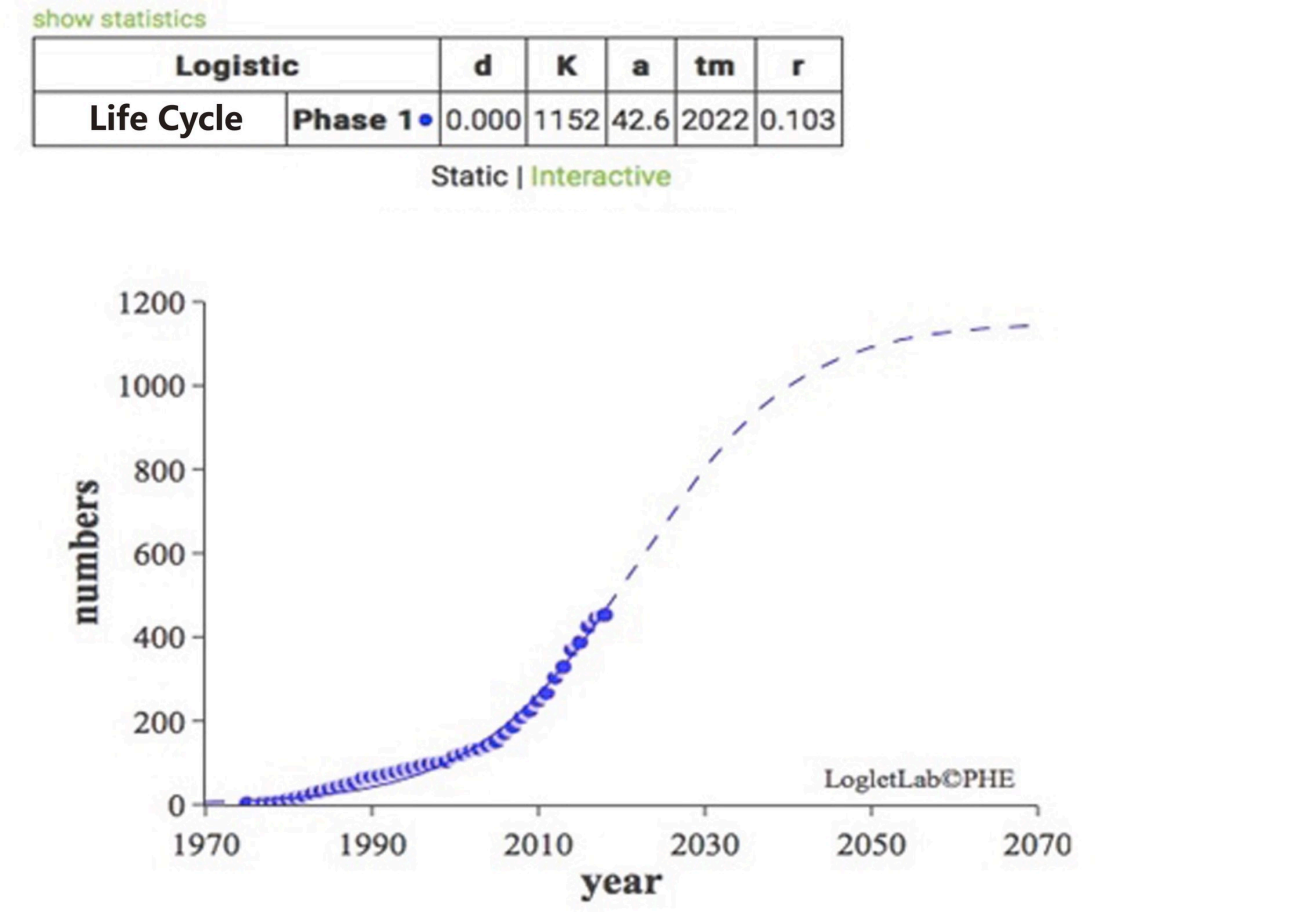
Technology development life cycle fit diagram.

The development of the technology conforms to the law of the S curve. By fitting the S-growth curve, the life cycle value of the ship-integrated power system can be calculated, the current growth stage of the technology can be accurately approximated, and the technical prediction can be made. This paper uses the Loglet Lab 4 software and the Logistic model equation to draw the technology development life cycle of the ship-integrated power system field through the patent accumulation and the numerical characteristics of the earliest patent application year in order to approximate the life cycle development stage of this field [19, 20]. The dot distribution in Fig 8 shows the scatter diagram of the patent accumulation number and the application year, and the curve is the logistic curve, which finally reaches the same points basically. The three data in the upper part of the diagram are the saturation point, turning point, and growth time analyzed by software. These three data were substituted into the formula in order to calculate the time inflection points: *t*1 = 1990, *t*2 = 2011, *t*3 = 2032, and *t*4 = 2053 [21, 22]. As the earliest retrieval of this patent in the Derwent database was in 1975, it can be concluded that the period 1975–1989 is the embryonic stage, which is also called the initial stage of technology. There are fewer research studies in this stage, the number of patents is small and unstable, and there are few research results as well. The period from 1990 to 2010 was termed a slow growth period. At this stage, the ship-integrated power system attracted adequate attention from all parties. The number of patents began to increase gradually, and the number of participants also increased. It is predicted that the period from 2011 to 2031 will be the rapid growth period, and the integrated power system may become the research hotspot in the direction of ships. The number of patents increases rapidly with an obvious upward trend. At present, patent research may be in the early stage of the rapid growth period. The period 2032–2053 is forecast as the mature period of technology. In this period, the technology will begin to mature, there will be a high degree of social recognition, patent accumulation growth will begin to slow down, technological innovation will become difficult, the core of technology will begin to concentrate in the hands of some patentees, and the enterprise will not be considered suitable to enter the field. Patent accumulation is forecast to peak at 1,152 in 2053, before recession beings in the field of technology [23].

- Enterprise Technology Concentration

With the help of the patented technology development life cycle, the overall development (453 records) is divided into three stages: the embryonic stage (1975–1989, 64 records), the slow growth stage (1990–2010, 185 records), and the rapid growth stage (2011–2019, 204 records) [24, 25]. The IPC classification number of patents in each stage is counted, and the first five technology branches in each stage are used for calculating the enterprise technology concentration degree [26].

(1) Analysis of the technology concentration in the embryonic stage. After sorting out 64 patents in the embryonic stage, a total of 31 technology branches are observed. The first five major technology branches and the number of patents that were applied are B63H (30), H02P (16), B63J (14), H02J (11), and B63B (7), with a technology concentration of 82.81%.

The statistics of the five IPC classification numbers and their subclasses in the germination stage are as follows: B63H primarily refers to the propulsion device or the steering device of a ship, which is related to the propulsion power equipment or device of a ship; H02P refers to the control or the adjustment of the motor, generator or electromechanical converter, control transformer, and reactor or choke, and focuses on the generator control device used to obtain the required output value; B63J refers to the auxiliary equipment on board, which focuses on the main design of the auxiliary equipment driving technology; H02J refers to the power supply or the distribution of the circuit device or the system and the energy storage system, which focuses on the ac inductance or the ac distribution network circuit device; B63B refers to ships or other water vessels and ships equipment, which is primarily related to the subject technology research.

These technology branches, which involve the basic technologies of ship-integrated power systems, such as propulsion power systems and distribution systems, developed rapidly at this stage and became active technology branches.

(2) Analysis of the technology concentration in the slow growth period. After sorting out 185 patents in the embryonic stage, there are 85 technology branches, and the first five major technology branches and the number of patents applied are, namely, B63H (100), H02P (35), B63J (33), H02K (26), and H02J (26), respectively, with a technology concentration of 75.31%.

In this stage, the number of patent applications has increased substantially, the technical field has expanded, and more and more industries participated in the industry of the ship-integrated electric power system. Old technologies have continued to mature, and new technologies have continued to emerge. The focus was on various technologies related to the development of the ship-integrated electric power system. It has been found that B63H, H02P, B63J, and H02J have not been significantly different from each other in the patented technology field and the embryonic stage according to the statistical analysis of the sub-technologies of these technologies in this stage, and studies are still being conducted on these technologies. The difference was in the H02K technology branch, which refers to the development of motor technology and focuses on technologies related to motor winding parts.

(3) Analysis of the technology concentration in the rapid growth period. After sorting out 204 patents in the embryonic stage, there are 68 technology branches. The first five major technology branches and the number of patents they applied for are, namely, B63H (121), B63J (70), B63B (51), H02J (47), and H02P (29), with a technology concentration rate of 87.25%.

At this stage of the ship-integrated power system, the rapid increase in the number of patent applications also gradually formed the core of the integrated power system technology, such as ship propulsion and steering gear, ship auxiliary equipment, as well as electric motors, generators or electromechanical converter control or adjustment, and the circuit of the power supply or the power distribution device or system and the energy storage system. Because of the importance and status of these technologies and their development, researchers are still interested in studying these technologies and obtaining new breakthroughs.

Although the technical research related to B63H and H02P has not changed much, B63J has gradually changed from the driving technology of auxiliary equipment to the research on technical topics not included in other groups under the IPC classification number, and the technical topics have gradually improved and extended to other peripheral-related technologies. B36B has focused more on the study of ships suitable for specialized purposes or similar floating structures. H02J has changed from the research of the circuit device to the research of the device technology used for the charging or depolarization of the battery pack or device technology used for the power supply from the battery pack to load. In recent years, adequate attention has been paid to the research of batteries.

To sum up, the technology concentration index of the ship-integrated power system basically remains above 50%, and in recent years, the trend of rising, ship-integrated power system has gradually established the core technology objectives. We suspect that, with the passage of time, the overall change in technical topics is not significant, but the study will be more in-depth, from the power system or device (push or steering gear, motor, power supply or power distribution system) and its auxiliary equipment to step by step its internal components and devices, such as electrical components and battery power system devices.

## 4. Conclusions

This paper analyzes the patent data related to the ship-integrated power system in the DII database and draws the following conclusions about the technical competitiveness of enterprises in this field.

By drawing the technical strength distribution in the field of the ship-integrated power system with the help of Derwent’s patentee code, CSHI-C has the largest number of patents, followed by fJIE-C, GLDS-C, GENE-C, WTNE-C, and DEWO-C codes.

The integrated power system at the core of the patent technology is roughly classified into the following five categories: (1) the internal structure of the electric propulsion system, (2) the structure design of the electric propulsion system, (3) a real-time monitoring system of the electric power system and automation devices and methods of the synchronous operation, (4) the drive for the power control, adjustment, and management, and (5) the structure of the machine and the control detection module design. The design, control, detection, and management of the electric propulsion system should be realized in order to meet the automation needs of the generation and distribution of the Marine electric power, and the focus should be on the design and control of the power equipment with the generator and motor as the core elements.

By using two measures of the patent quantity and the patent quality, we identified the dominant and productive firms in this field. The superior enterprises have a large number of patents with high quality, and the representative is the General Electric of the United Kingdom, whose core field is the transformation of the generator system. High-yielding firms, represented by the Daewoo Shipbuilding and Marine Engineering and Fuji Electric of Japan, also have a core field of propulsion, but the latter has also performed well in shipboard circuits.

At present, the integrated power system and its related core technologies are in the rapid growth stage. It is anticipated that the related technology patents will increase rapidly in the next few years, and more and more enterprises will participate in this field [23]. The application time of 453 core patents can be roughly divided into the following three development stages: the embryonic stage (1977–1989), the slow growth stage (1990–2010), and the rapid growth stage (2011–2019). Based on the embryonic stage to the rapid growth stage, ship propulsion or steering units, auxiliary equipment on board, as well as motors and power supply and distribution systems have been considered as the technical areas of concern, especially the gradual refinement of internal components and circuit configurations from ship propulsion units or devices. In addition, the research studies have changed from circuit devices to battery packs, and the field of technical research has also changed with the development of the times in the rapid growth period.

Though the technology field intensifies the development, some advantageous enterprises also grow hand in hand. From the perspective of the overall development trend, Daewoo Shipbuilding and Ocean Engineering Co., Ltd., and Hyundai Heavy Industries Group Co., Ltd., of South Korea, as the late show, grabbed the opportunity and quickly established their own advantages in the rapid growth period. Meanwhile, the Fuji Electric and Nishichi Electric Co. of Japan, the Siemens Ag of Germany, and the Samsung Heavy Industries Co. of South Korea have steadily built up their strengths in different sectors. However, the apparent lack of momentum of General Electric, Toshiba, and Mitsubishi Heavy Industries during the rapid growth period caused an increase in the number of potential competitors that entered the sector and increased the competitive pressure.

In general, the field of the ship-integrated power system technology is highly competitive and has great potential for development. Japan and Korea have emerged as strong competitors in this field. The ship-integrated power system has entered a period of rapid growth. Although the technical core field is in the embryonic stage, there is still a large scope for development and great potential for technological innovation. In this regard, researchers can pay attention to the patented technologies of Japanese and Korean companies and explore new avenues in the latest frontier fields when developing integrated power systems. At the same time, the patent of European and American enterprises can be studied, especially the General Electric of the United Kingdom and the Siemens Co. of Germany, in order to explore the core research area and technical background of the integrated power system in Europe and America and avoid the deviation in the R&D direction.
